# Coincidence of follicular lymphoma and hurtle cell thyroid carcinoma in a patient at presentation: which one is the source of bone metastasis? Case report and review of the literature

**DOI:** 10.1002/ccr3.942

**Published:** 2017-04-12

**Authors:** Hasan Atilla Ozkan, Arda Akoluk, Turhan Ozler, Isin Dogan Ekici, Nalan Alan Selcuk

**Affiliations:** ^1^ Department of HematologyYeditepe School of MedicineIstanbulTurkey; ^2^ Yeditepe School of MedicineIstanbulTurkey; ^3^ Department of General OrthopedicsYeditepe School of MedicineIstanbulTurkey; ^4^ Department of PathologyYeditepe School of MedicineIstanbulTurkey; ^5^ Department of Nucleer MedicineYeditepe School of MedicineIstanbulTurkey

**Keywords:** BMT, clinical decision making, follicular lymphoma, positron emission tomography with computed tomography, thyroid incidentaloma

## Abstract

Thyroid incidentaloma is defined as a new identified thyroid lesion occasionally detected during imaging studies. Incidence of thyroid incidentalomas is relatively rare in patients with lymphoma. Because of high rate of malignancy, these lesions with high intensity focal ^18^ FDG uptake detected on positron emission tomography with computed tomography (PET/CT) should undergo to biopsy regardless of size.

## Introduction

Thyroid incidentaloma is defined as a newly identified thyroid lesion occasionally detected during imaging studies. Typically, these lesions are not palpable and have not been noted on routine physical examination 1, 2. The ^18^F‐Fluorodeoxyglucose positron emission tomography with computed tomography (FDG‐PET/CT) has been widely used for the localization, staging, prognosis, and follow‐up of various types of malignancies. It is also considered the imaging method of choice for the detection, pretreatment staging, and response assessment of many lymphoma subtypes 3.

The normal thyroid gland shows very low‐grade ^18^FDG uptake 4. Occasionally, a significant ^18^FDG uptake in the thyroid gland is identified as an incidental finding. Focal thyroid uptake has been reported in 1.2–4.3% of patients with cancer and healthy subjects with a risk of malignancy ranging from 14% to 50% 5.

Here, we report the case of an 65‐year‐old male patient who presented with pathologic vertebral fracture, and then diagnosed as follicular lymphoma with multiple bone metastasis and at the same time with thyroid follicular carcinoma with hurtle cell variant which was incidentally detected on FDG‐PET/CT imaging performed for staging of follicular lymphoma (FL).

## Case Report

Sixty five ‐year‐old male was referred to our institution from a community hospital with a two‐month history of backache that later was found to be caused by third lumbar vertebra compression fracture. Physical examination was completely unremarkable except for right posterior cervical lymphadenopathy around 2 cm, no hepatomegaly or splenomegaly was detached and he had no B symptoms. Vertebroplasty pathology results came back as lymphocytes with polymorphonuclear leukocytes; however, excisional lymph node biopsy revealed Grade 2 FL (Fig. 2A and B).

**Figure 1:**
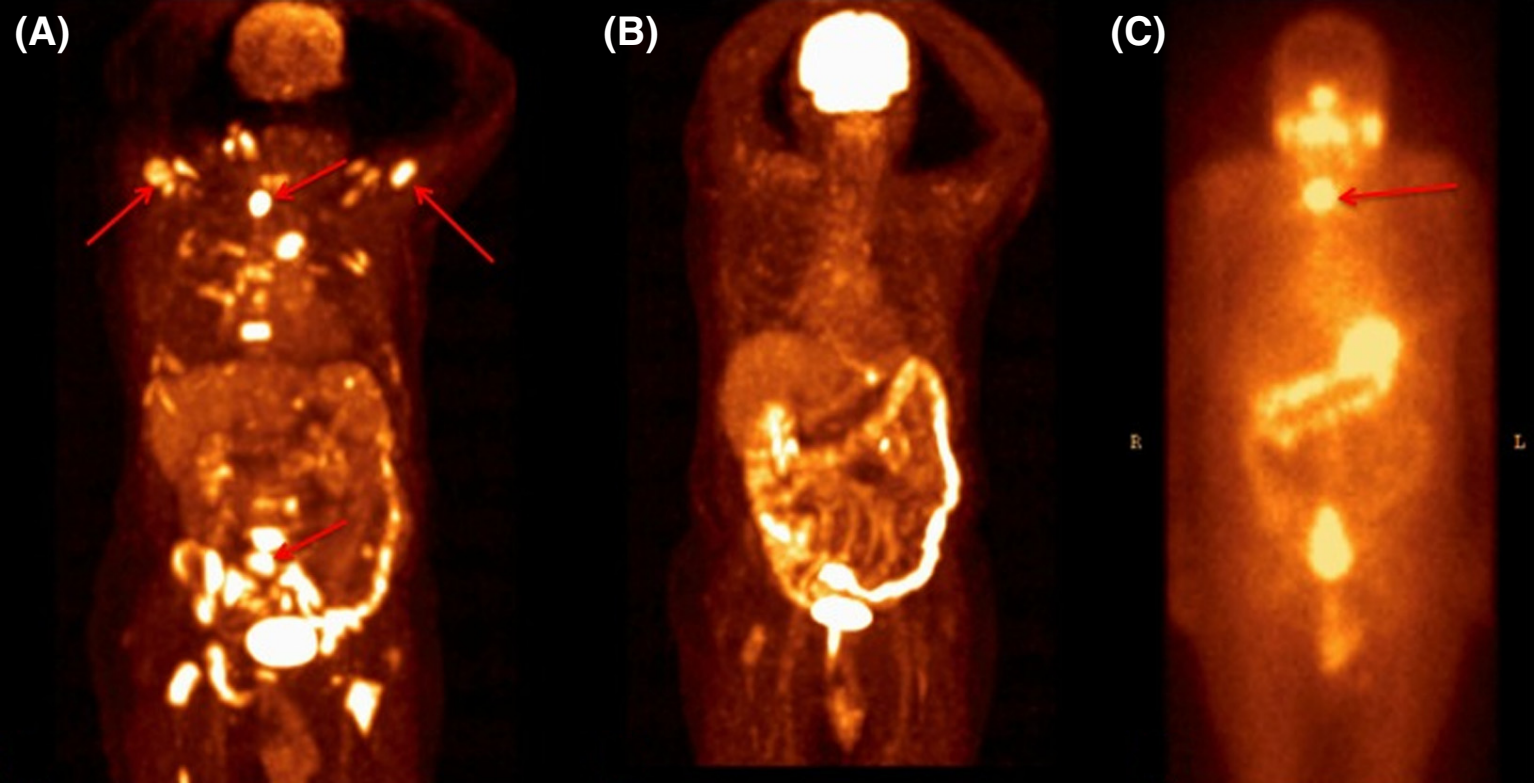
FDG‐PET MIP image. (A) FDP‐PET image showing thyroid lesion, bone metastasis, and lumbar vertebra involvement(arrows),before chemotherapy. (B) Control FDG‐PET image after treatment showing full remission. (C) Whole body image after 200 mCI I131 treatment showing only thyroid involvement,arrow showing thyroid involvement.

FDG‐PET/CT performed for FL staging showed multiple bone metastasis, cervical and supramediastinal lymph nodes with maximum SUV of 7. Focal uptake in right thyroid lobe with SUV of 30 and hypermetabolic area in right rosenmuller fossa were also detected (Fig. 1A).

**Figure 2:**
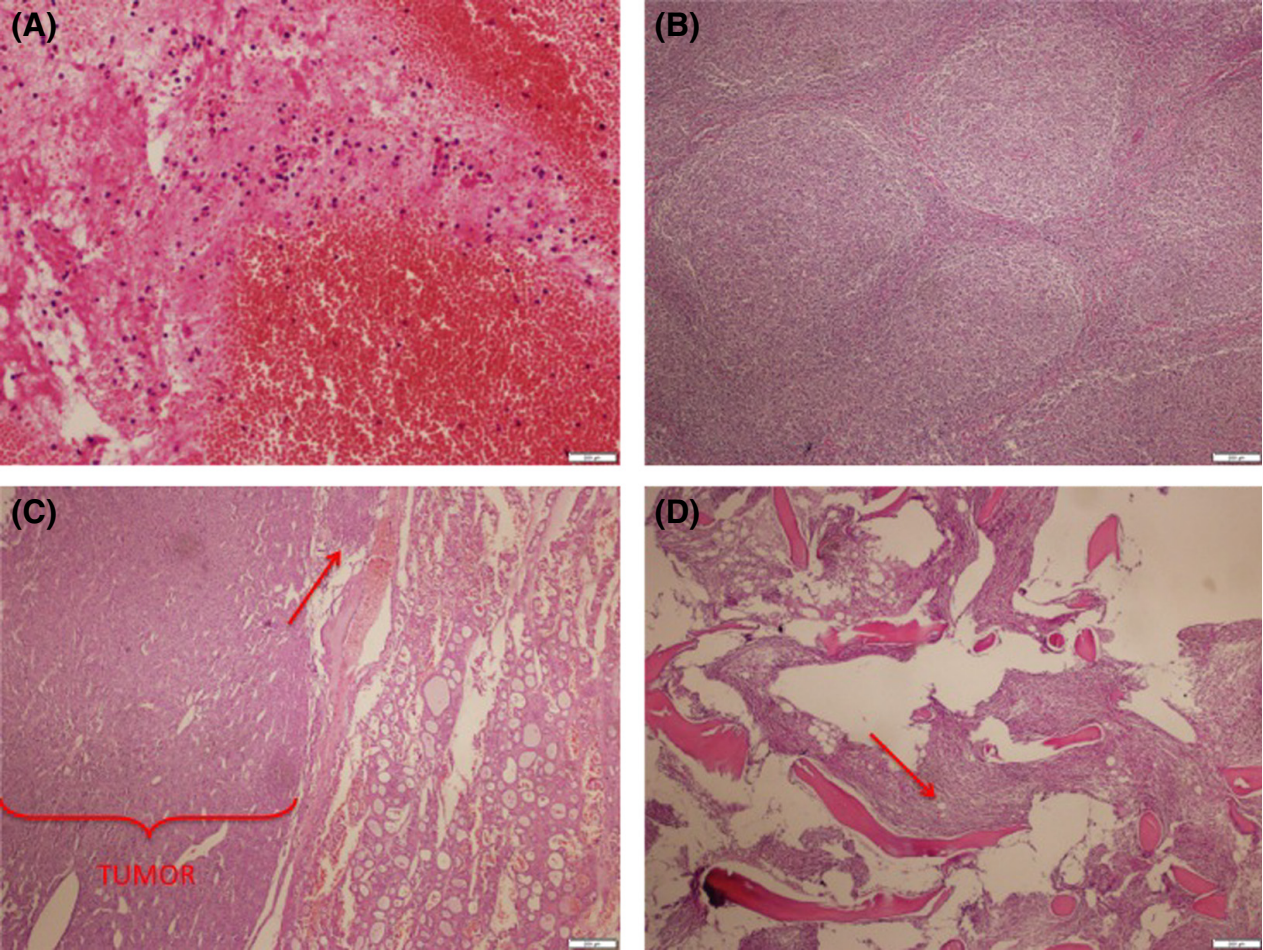
Pathology images. (A) L3 vertebra aspiration and biopsy pathology results showing inflammatory. H&E x40. (B) Cervical lymph node biopsy results showing follicular atypical lymphoid cells. H&E x40. (C) Thyroid surgical material showing follicular carcinoma with hurtle cell variant.Thyroid capsule invasion is shown in arrow. H&E x40. (D) Right humerus bone marrow biopsy showing atypical lymphoid cell infiltration. Arrow shows bone metastasis. H&E x40.

Thyroid function tests were within normal limits. Given the high focal 18FDG uptake of thyroid nodule, fine needle biopsy was performed to rule out transformation to high‐grade lymphoma. The biopsy reported as thyroid follicular carcinoma with hurtle cell variant, which was also confirmed by total thyroidectomy pathology specimen (Fig. 2C).

Following total thyroidectomy postoperative radioactive I131 treatment was performed. Control FDG‐PET/CT revealed persistent bone metastasis with resolution of thyroid nodule (Fig. 1C). After long discussions with multidisciplinary team,bone biopsy was performed to determine primary source of the bone lesions. Biopsy obtained from the bone lesion was consistent with FL involvement (Fig. 2C and D).

At the end of the evaluation, the patient was put on chemoimmunotherapy with R‐CHOP regimen. After four cycles, interim FDG‐PET/CT showed no residual disease and no thyroid uptake. (Fig. 1B). Then, he received two more cycles of R‐CHOP and followed by rituximab maintenance therapy. The patient is event‐free for one year.

## Discussion

Here, we report the second case of incidental thyroid follicular carcinoma with hurtle cell variant together with FL. In addition to that, the presence of bone metastases at the time of diagnosis made it challenging for us to determine the primary origin of the bone lesions. This makes our case unique in the literature.

A retrospective study by Pagano et al. 6 demonstrated that the prevalence of incidental thyroid ^18^FDG uptake was nearly 1.76%. The presence of focal uptake with high SUV max (>5) and euthyroidism correlated with high‐likelihood of malignancy. This is consistent with our patient who had normal thyroid function and a focal 18FDG uptake with SUV of 30 max on his PET scan.

Follicular lymphoma is the second most frequent lymphoma subtype worldwide, and its incidence has been nearly doubled with the past three decades, especially in Western countries 7. FDG‐PET/CT has been routinely used in the FL assessment.It is also useful in cases where transformation to high‐grade lymphoma is suspected by targeting the biopsy to lesion with highest FDG uptake 8. The risk of histologic transformation of FL has been reported to range from 1.4% to 4.4% per year 9. Our case, indeed, received fine needle biopsy of thyroid lesion to exclude histological transformation of FL to high‐grade lymphoma. Although incidental thyroid ^18^FDG uptake has been reported in 1.2–4.3% in the literature, its incidence is extremely rare in lymphoma patients with only about 10 well documented cases reported in the English literature (Table 1) 5.

**Table 1:** Case reports and series in the literature

Primary cancer	Age/Sex	Suv value	Tumor size (cm)	Histology	Authors
Hodgkin's Lymphoma	64/F	3.2	15	Hyperplastic follicular benign nodule (Biopsy with suspicious for Hurtle	Jamsek et al. 12
Hodgkin's Lymphoma	67/M	6.2	Not given	Papillary thyroid carcinoma	Bonabi et al. 13
Lymphoma	63/M	6.3	18	Papillary thyroid carcinoma	Kim et al. 14
Lymphoma	64/F	2.9	18	Indeterminate	
Diffuse B‐cell lymphoma	80/F	NG	NG	Papillary thyroid carcinoma	Ishimori et al. 15
Follicular lymphoma	59/M	NG	NG	Papillary thyroid carcinoma	
Lymphoma	NG	NG	2	Papillary thyroid carcinoma	Jin et al. 16
Non‐Hodgkin's Lymphoma	74/F	5.6	12	Papillary classical variant	Pagano et al. 6
Hodgkin's Lymphoma	75/F	6.9	9	Papillary classical variant	
Hodgkin's Lymphoma	66/F	11.8	16	Papillary follicular variant	
Follicular High grade non‐Hodgkin's Lymphoma	64/M	14	8.5 × 4.2	Hurtle cell variant carcinoma	Makis et al. 11
Follicular High grade non‐Hodgkin's Lymphoma	65/M	7	4	Hurtle cell variant carcinoma	This case

NG, Not Given; F, Female; M, Male; SUV, Standardized Uptake Value.

Having bone lesions of our case also puzzled us in differential diagnosis deciding whether it stem from thyroid carcinoma or FL. Persistent ^18^FDG uptake of the bone lesions following radioactive I131 treatment pushed us to perform histologic examination from one of the bone lesions to confirm the exact diagnosis. And, biopsy obtained by surgical procedure resulted in another unexpected condition with bone metastasis of FL, which rarely represent with bone metastasis 10.

To the best of our knowledge, there are only ten cases describing lymphoma with thyroid incidentaloma in the literature (Table 1). (6,11,12,13,14,15,16) Only one of them was FL patient with thyroid hurtle cell variant carcinoma 11. Others were either papillary cell carcinoma or undetermined. Almost all cases were over 60 years old and mortality rate was high considering the aggressiveness of this type of tumor. There was no tendency for gender even though the only other FL case with Hurtle cell variant was also male. Tumor size ranged from 2 to 16 cm, in our case tumor size was relatively smaller around 4 cm. SUV ranged from 2.9 to 14 making it a less useful tool to predict aggressiveness of thyroid incidentalomas. In our case, intensity of SUV was 30 making it one of the highest SUV reported in the literature. There were only three FL cases with coincidental thyroid lesion. Only one of them had high‐grade FL. That case had no metastasis and only presented with thyroid incidentaloma found in FDG‐PET/CT scan. However, in our case, we had to struggle to decide whether bone metastasis stem from thyroid carcinoma or FL making management approach more difficult.

In conclusion, although incidence of thyroid incidentalomas are relatively rare in patients with lymphoma, because of high rate of malignancy described in the literature, these lesions with high intensity focal ^18^FDG uptake detected on PET/CT scan should undergone to biopsy regardless of size. This is also essential for making differential diagnosis whether it is a histological transformation of the underlying lymphoma or a thyroid carcinoma due to needing different management strategies.

## Authorship

HAO and AA: wrote the manuscript. HAO and AA: collected the data. HAO, AA, TO, IDE, NAS: took care of the patients. All Authors approved and critically helped revision of the manuscript.

## Conflict of Interest

The Authors declare no conflicts of interest.
